# Identification of a serum-induced transcriptional signature associated with metastatic cervical cancer

**DOI:** 10.1371/journal.pone.0181242

**Published:** 2017-08-30

**Authors:** Anna Palatnik, Shuyun Ye, Christina Kendziorski, Marissa Iden, Jessica S. Zigman, Martin J. Hessner, Janet S. Rader

**Affiliations:** 1 Department of Obstetrics and Gynecology, Medical College of Wisconsin, Milwaukee, Wisconsin, United States of America; 2 Department of Biostatistics and Medical Informatics, UW-Madison, Madison, Wisconsin, United States of America; 3 Department of Pediatrics, Max McGee National Research Center for Juvenile Diabetes, Children’s Research Institute, Children’s Hospital of Wisconsin, Medical College of Wisconsin, Milwaukee, Wisconsin, United States of America; Universitat des Saarlandes, GERMANY

## Abstract

**Objective:**

Tumor cells that escape local tissue control can convert inflammatory cells from tumor suppressors to tumor promoters. Moreover, soluble immune-modulating factors secreted from the tumor environment can be difficult to identify in patient serum due to their low abundance. We used an alternative strategy to infer a metastatic signature induced by sera of cervical cancer patients.

**Methods:**

Sera from patients with local and metastatic cervical cancer were used to induce a disease-specific transcriptional signature in cultured, healthy peripheral blood mononuclear cells (PBMCs). An empirical Bayesian method, EBarrays, was used to identify differentially expressed (DE) genes with a target false discovery rate of <5%. Ingenuity Pathway Analysis (IPA) software was used to detect the top molecular and cellular functions associated with the DE genes. IPA and *in silco* analysis was used to pinpoint candidate upstream regulators, including cancer-related microRNAs (miRNAs).

**Results:**

We identified enriched pathways in the metastatic cervical group related to immune surveillance functions, such as downregulation of engulfment, accumulation, and phagocytosis of hematopoietic cells. The predicted top upstream genes were IL-10 and immunoglobulins. *In silco* analysis identified miRNAs predicted to drive the transcriptional signature. Two of the 4 miRNAs (miR-23a-3p and miR-944) were validated in a cohort of women with local and metastatic cervical cancer.

**Conclusions:**

This study supports the use of a cell-based assay that uses PBMC “reporters” to predict biologically relevant factors in patient serum. Further, disease-specific transcriptional signatures induced by patient sera have the potential to differentiate patients with local versus metastatic disease.

## Introduction

The most significant prognostic factor for poor survival rates in patients with invasive cervical cancer is metastasis [1]. Unfortunately, metastasis can be difficult to identify and treat, and it does not directly relate to tumor size. Some patients with stage I disease, and even some with small and seemingly localized tumors, have metastases identified in lymph nodes or distant organs [1]. Consequently, identifying biologic pathways and biomarkers through novel techniques could improve detection and treatment for women with cervical cancer.

Metastasis is a complex process involving both intrinsic changes in tumor cells and the host response. For example, cancer cells can metastasize by exploiting a large variety of immune-mediated escape mechanisms [2]. They can secrete growth factors and cytokines to create a state of chronic inflammation that prompts clonal expansion of myeloid precursors and directs their movement to the primary tumor environment or metastatic sites [3]. In their new locations, these cells can alter the tissue microenvironment and promote the recruitment and colonization of tumor cells to form macrometastases. Tumor cells can also shed or restrict the presentation of ligands involved in their recognition by natural killer (NK) cells or cytotoxic T lymphocytes (CTLs) [2]. Circulating factors identified in the blood during disease progression include proteins (cytokines), microRNAs (miRNAs), and cell-free tumor and viral DNA. Many of these factors may be of low abundance and difficult to detect through global discovery techniques. Therefore, detection of potentially more accurate, yet low in abundance, serum biomarkers remains a challenge.

The objective of the current study was to identify a transcriptional response signature of metastatic cervical cancer induced by serum factors, providing information on the overall state of immune pathways and potential biomarkers of metastatic disease. We adapted a novel cell-based assay to identify transcriptional signatures in peripheral blood mononuclear cells (PBMCs) induced by serum from cervical cancer patients with local or metastatic disease. PBMCs contain a breadth of surface receptors due to their varied cell populations of T and B lymphocytes, monocytes, NK cells, and dendritic cells. This assay was previously shown to be sensitive and accurate for detecting inflammatory biomarkers that predict type 1 diabetes as well as characterizing inflammation associated with inflammatory bowel disease and disorders characterized by airway infection and inflammation [4, 5]. Using sera from patients with squamous cell carcinoma (SCC) of the cervix, we identified a gene expression signature for metastatic cervical cancer. These gene signatures were used to deduce upstream regulators, using pathway and *in silico* analysis. Our data suggest that this method of measuring alterations in gene expression induced by patients’ sera has the potential to predict immune status in the patient and to identify metastatic disease-specific biomarkers.

## Material and methods

### Subject characteristics

We used serum specimens from women diagnosed with localized or metastatic cervical SCC. Samples were obtained from each participant at the time of cancer diagnosis but before treatment and were stored at -80° Celsius, after informed consent was obtained. The patient data collected included age, race, metastasis (determined by FDG-PET and/or pathology), staging, recurrences, and survival status. The study consisted of 10 subjects diagnosed with cervical SCC (Table 1); 5 women had local disease without nodal involvement and were disease-free at least 18 months following diagnosis, while the other 5 had distant disease (positive by PET-CT) or metastatic foci in lymph nodes confirmed by pathology at the time of diagnosis. Sera from subjects in the initial assays plus sera from 39 additional patients were used in miRNA validation experiments (Table 2). The study protocol was approved by the institutional review board at the Medical College of Wisconsin.

**Table 1:** Subject characteristics for reporter assay.

	Age	Race	Metastatic sites by PET-CT scan at diagnosis	Stage	Pathologic metastatic sites at diagnosis	Alive or dead	Months from diagnosis
Localized cancer							
	19	Caucasian	Negative	Ib2	Negative pelvic lymph nodes	Alive	130
	49	Caucasian	Negative	Ib2	Negative pelvic lymph nodes	Alive	52
	38	Caucasian	Negative	Ib1	Negative pelvic lymph nodes	Alive	18
	43	Caucasian	Negative	Ib1	Negative pelvic lymph nodes	Alive	108
	35	Caucasian	Negative	Ib2	Negative pelvic lymph nodes	Alive	106
Metastatic cancer							
	31	Caucasian	Pelvic and aortic lymph nodes positive	Ib1	Pelvic and aortic lymph nodes	Dead	6
	44	African American	Pelvic and aortic lymph nodes positive	Ib2	Pelvic and aortic lymph nodes	Alive	108
	31	Mixed race	Pelvic and aortic lymph nodes positive	Ib2	Pelvic and aortic lymph nodes	Dead	48
	26	Caucasian	Pelvic and aortic lymph nodes positive	Ib2	Not done	Dead	5
	38	Caucasian	Negative	Ib1	Pelvic lymph nodes	Alive	108

* PET-CT scan: positron emission tomography (PET) and computerized tomography (CT)

**Table 2:** Subjects characteristics for miRNA assay.

Characteristic		Lymph node positive	Lymph node negative
Mean age (y)		46.5	48.5
Race	Caucasian	14	18
	African American	5	2
Stage	Ib1	12	16
	Ib2	6	4
	Iib	1	0
	Total	19	20

### PBMC cultures and gene expression analysis

Gene expression was induced by culturing commercially cryopreserved PBMCs from a single healthy donor (UPN727; Cellular Technology Ltd, Shaker Heights, OH) for 9 h at 37°C in 5% CO_2_ with media supplemented with sera of cancer patients from each of the 2 groups (local or metastatic), as previously described by Hessner et al. [4, 5]. The composition of the UPN727 cell by a standard flow panel was described in a previous publication and was found to be in the “normal range” [6]. The post-thaw viability of UPN727 is >90%, and multiparameter flow cytometry has shown that the relative abundance of NK cells, CD8+ T cells, CD4+ T cells, B cells, and monocytes does not differ significantly from the normal ranges expected for fresh PBMCs [5]. The cultures were prepared in a Costar 24-well plate (Corning, St Louis, MO), using 12 wells per condition (500,000 cells/well in 300 μl of RPMI 1640 medium plus 200 μl of patient-derived serum). In addition to the 5 serum samples from patients with non-recurrent local disease and the 5 samples from patients with metastatic cervical SCC, we created an 11th co-culture that contained cancer-free human serum as a control.

PBMCs of each co-culture were recovered by centrifugation and total RNA was extracted using TRIzol reagent (Life Technologies, Grand Island, NY). Complementary DNA (cDNA) was synthesized from purified RNA (~50 ng) using an Affymetrix two-cycle cDNA synthesis kit (Affymetrix, Santa Clara, CA). cDNA was then labeled and fragmented before being hybridized (in accordance with the Affymetrix GeneChip expression analysis technical manual) to a GeneChip Human Genome U133 plus 2.0 array, which interrogates ~47,000 transcript/transcript variants. RNA from each culture was independently analyzed. The study methods are summarized in S1 Fig.

In silico analysis of differentially expressed (DE) genes to identify potential upstream miRNAs.

To identify candidate miRNAs, we performed *in silico* analyses using starBase [7], TargetScan [8], and DIANA-mirExTra [9]. Each of these methods identifies regulatory interaction networks among multiple classes of RNA. Candidate miRNAs that were identified by any of these methods were considered in further analysis. The top 20 DE genes (Table 3) were entered into starBase and TargetScan to identify their potential regulatory miRNAs. The top 383 DE genes were uploaded into DIANA-mirExTra, an algorithm that predicts the effects of miRNAs on expression levels of protein-coding transcripts based on the frequency of 6-nucleotide motifs (hexamers) in the 3'UTR sequences of our DE genes. The resulting list of miRNAs was pared down to the top overlapping candidates identified from the three *in silico* methods. Diana-mirExTra analysis ranked miRNA candidates from DE genes of the entire 383 gene list. Four miRNAs (miR-23a-3p, miR-23b-3p, miR-193b-5p and miR-944) were chosen for further examination with quantitative polymerase chain reaction (qPCR) as described below.

**Table 3:** Most significantly up- and downregulated genes in metastatic cancer from reporter assay.

Upregulated genes	Fold change up	Downregulated genes	Fold change down
*SIAH1*	2.900	*ANXA1*	-2.703
*TEP1*	2.860	*MS4A4A*	-2.632
*POLK*	2.720	*ADORA3*	-2.500
*ATM*	2.660	*FPR1*	-2.326
*LPP*	2.660	*MRC1*	-2.273
*CXCL11*	2.450	*GPR34*	-2.273
*IBTK*	2.410	*WAC*	-2.174
*STK17A*	2.380	*CD163*	-2.174
*CXorf56*	2.360	*VCAN*	-2.041
*GNL3L*	2.320	*VNN1*	-2.000

FDR <0.05, and largest fold change

### Quantitative PCR

RNA from serum (400 μl) was isolated using the miRCURY RNA Isolation Kit—Biofluids (Exiqon, Woburn, MA) and reverse-transcribed into cDNA, using target-specific primers supplied with corresponding Taqman probes (Life Technologies, Inc) for miRNAs miR-23a-3p, miR-23b-3p, miR-193b-5p and miR-944. The qPCR was run with the following thermal cycling conditions: enzyme activation for 10 min at 95°C, followed by denaturing and annealing, 95°C for 15 s and 60°C for 60 s, respectively, for 40 cycles. Each reaction was run in triplicate using U6 for normalization. All data were analyzed using the ΔCt method.

### Statistical analysis

All PBMC assay analyses were carried out in R [10], a publicly available statistical analysis environment. The packages affy and EBarrays are available at Bioconductor, an on-line suite of tools for analyzing genomic data [11]; R package allez 1.0 is also publicly available [12].

Pre-processing and normalization: The Affymetrix probe data were processed using Robust Multi-Array Analysis (RMA), as implemented in the Affymetrix package affy, to obtain normalized summary scores of expression for each probe set on each array [13]. RMA fits a linear model to the log probe intensities for each probe set. The linear model includes a sample effect (the parameter of interest), a probe effect, and an error term. The standard errors are normalized, setting the same median standard error across arrays. The normal control chip was included in the normalization, though not involved in further analysis.

Identification of DE genes: EBarrays was used to identify probe sets that were differentially expressed between sera from women diagnosed with localized or metastatic cervical cancer [12, 14]. EBarrays is an empirical Bayes approach that models the probability distribution of a set of expression measurements. It accounts for differences among genes in their true underlying expression levels, measurement fluctuations, and distinct expression patterns for a given gene among conditions [14]. The fitted model is used to assign probability distributions to every gene; each gene-specific distribution provides the posterior probability of that gene being DE. Thresholds were chosen to target a false discovery rate (FDR) of <5%.

Tests for enrichment: Tests for enrichment of common function among sets of DE genes were carried out using data from the Gene Ontology (GO) annotations and the Kyoto Encyclopedia of Genes and Genomes (KEGG). In Gene Ontology, transcripts (i.e., genes) are categorized at varying levels of biological detail; the three broadest levels are molecular function, cellular component, and biological process. The R package allez was used to test enrichment for each Gene Ontology category and KEGG pathway. In general, the interpretation of *p* values resulting from enrichment tests is not straightforward due to the many dependent hypotheses tested. Furthermore, the enrichment test tends to produce small *p* values when groups with few transcripts are considered. The statistical methods underlying allez adjust for these factors, providing increased power and sensitivity for identifying biologically meaningful sets [12].

Pathway and upstream regulation analysis: Ingenuity Pathway Analysis (IPA) software (Ingenuity systems, Redwood City, CA) was used to predict upstream transcriptional regulators of DE genes. *P* values were calculated using Fisher’s exact test to find statistically significant overlap between the genes in our dataset and genes that are controlled by transcriptional regulators. Following that, IPA software calculated an activation z-score, which determines whether an upstream transcriptional regulator has significantly more activated predictions (z>0) than inhibited predictions (z<0). The primary purpose of the activation z-score is to infer the activation states of predicted transcriptional regulators.

Finally, miRNA qPCR data were plotted and analyzed (unpaired *t* test) using GraphPad Prism version 6.0 (La Jolla, CA).

## Results

We used sera from women with localized (n = 5) or metastatic (n = 5) cervical SCC to drive transcription in healthy PBMC “reporters” and read the responses using genome-wide expression profiling. The transcriptional responses induced by the 11 (10 cancer and 1 healthy control) experimental samples were normalized using Robust Multi-Array Analysis (RMA) to obtain summary scores of expression for each profile set on each array [13]. We then used EBarrays to identify genes whose expression differed when comparing PBMCs stimulated by serum from women with localized versus metastatic disease. A total of 557 differentially expressed probe sets that mapped to 382 genes (of which 323 were unique) exhibited DE with a FDR of <5%. In the unique gene set, 184 were upregulated and 139 were downregulated in serum from women with metastatic disease. The most significant upregulated and downregulated genes from the patients with metastatic cervical cancer are shown in Table 3.

The top GO annotations included response to interferon-alpha and lipoteichoic acid (LTA), a major constituent of the cell wall of gram-positive bacteria, while the top KEGG pathway inferred was *Staphylococcus aureus* (*S*. *aureus*) infection (S1 Table). Core analysis was performed with IPA software to identify the most significant biologic functions associated with the genes that were more highly upregulated or downregulated by sera from metastatic patients (Table 4). Inflammatory response, phagocytosis, engulfment, and movement of blood cells were all decreased, with z-scores greater than −2.0 (Table 4). We used IPA upstream regulation analysis to identify candidate genes and signaling intermediates that could account for the observed serum-induced signatures. Overall, the most significant findings pointed to transcriptional inhibition by IL-10 (z = -2.094) and activation by immunoglobulin (z = 2.919) (Table 5).

**Table 4:** Top biological functions in the metastatic cervical cancer group.

Function annotation	*P*-value	Activation Z-score
Inflammatory Response	1.73E-15	-2.454
Immune Response of cells	1.02E-09	-2.527
Phagocytosis of blood cells	2.11E-09	-2572
Engulfment of blood cells	3.56E-09	-2.908
Phagocytosis of leukocytes	4.69E-09	-2.381
Engulfment of myeloid cells	5.68E-09	-2.513
Engulfment of cells	5.94E-09	-2.759
Cell movement of myeloid cells	6.90E-09	-2.133
Engulfment of leukocytes	8.10E-09	-2.727
Accumulation of neutrophils	1.10E-08	-2.711
Phagocytosis	1.19E-08	-2.927
Engulfment of phagocytes	1.30E-08	-2.403

**Table 5:** Top upstream regulators of dataset genes in metastatic cervical cancer.

Top upstream regulators	*P*- value	Activation Z-score
*IL10*	1.01E-16	-2.094
Immunoglobulin	4.95E-15	2.919
*IRF7*	7.38E-14	2.394
*PRL*	3.52E-13	2.488
*IFNB1*	4.09E-11	2.877
*IFNL1*	2.44E-09	3.251
*IL6*	2.60E-09	-2.903
*CSF3*	3.53E-09	-2.925
*IRF5*	4.38E-09	2.762
*DDX58*	5.50E-09	3.078
*IFNA2*	8.31E-09	3.821

**Fig 1:**
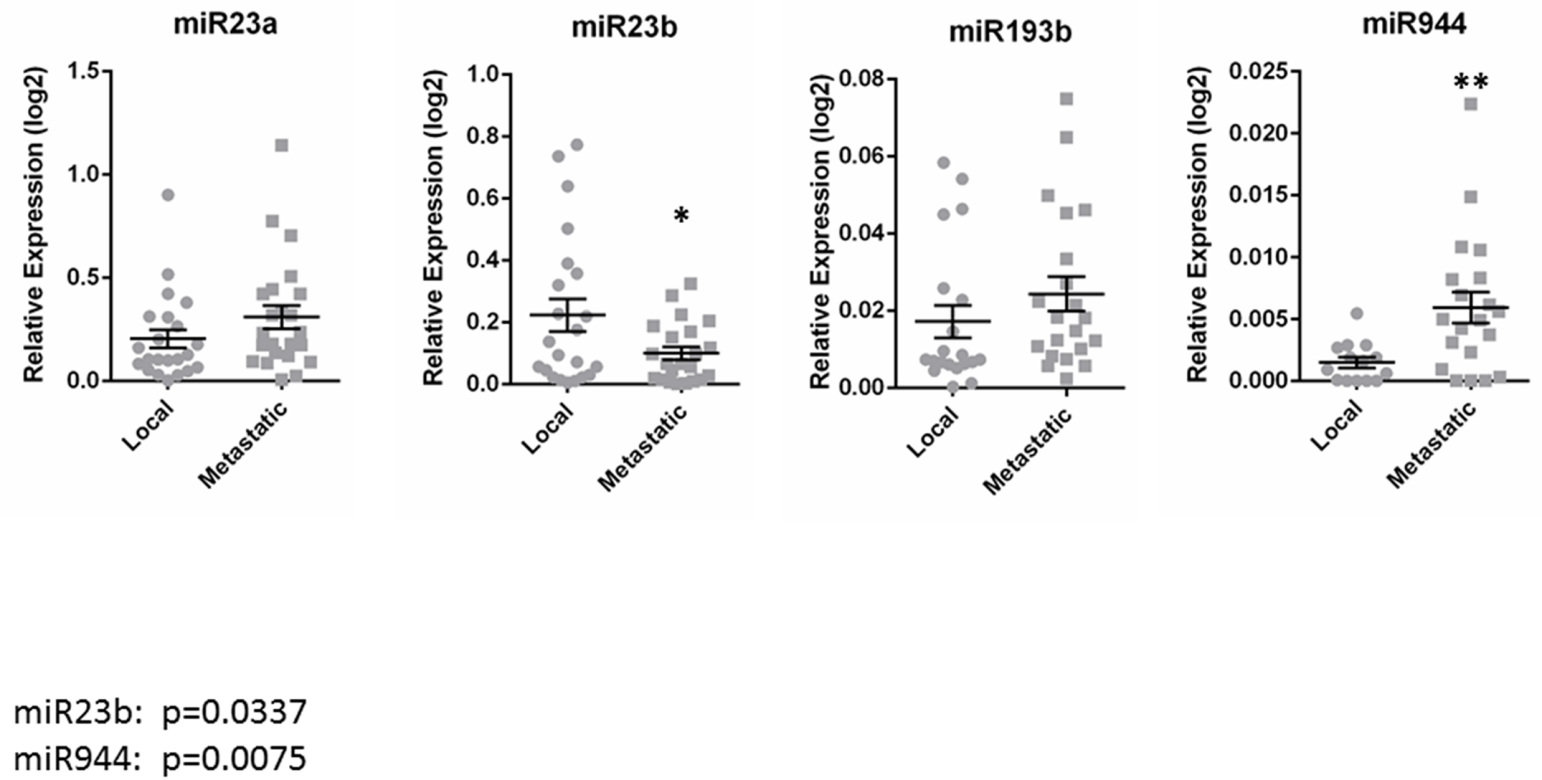
miRNA expression levels in serum from cervical cancer patients with local or metastatic disease. Expression levels of four candidate miRNAs were determined via Taqman-based qPCR using RNA extracted from the serum of patients with local (n = 25) or metastatic (n = 24) cervical cancer. Serum expression levels of miR-23b-3p were significantly lower in the metastatic group (B; *p<0.05), while miR-944 expression was significantly higher in serum from patients with metastatic disease (D; **p<0.01). Serum expression levels of miR-23a-3p (A) and miR-193b-5p (C) were not significantly different between the local and metastatic groups.

Finally, since miRNAs are abundant and stable in serum and are greatly dysregulated in cancer [15], we sought to determine if the observed DE genes could be explained by differences in serum miRNA composition of patients with local disease versus metastatic disease. *In silico* analysis revealed 4 candidate miRNAs (miR-23a-3p, miR-23b-3p, miR-193b-5p and miR-944) that are known to bind to and inhibit the expression of more than one of the DE genes listed in Table 2. Serum samples from patients with local or metastatic disease (Tables 1 and 2) were then analyzed with qPCR to evaluate the expression of these 4 miRNAs. Of these 4 candidate miRNAs, expression levels of 2 were found to be significantly different in serum from patients with metastatic versus local disease. Expression of miR-23b-3p was significantly lower (*p* = 0.0337), while miR-944 expression was significantly higher (*p* = 0.0075), in serum samples from 25 women with metastatic disease than in the 24 samples from women with localized disease (Fig 1).

## Discussion

The current study adopted a novel cell-based assay that used reporter PBMC cultures to characterize transcriptional signatures induced by serum from women with cervical cancer. Collectively, the results suggest that factors present in serum from women who have metastatic cancer rather than local disease associate with downregulation of immune-surveillance functions such as engulfment, accumulation, and phagocytosis of hematopoietic cells. Enrichment and pathway analyses suggest that the top candidates driving the serum effects include IL-10, immunoglobulin, and cellular responses to LTA and *S*. *aureus*. Although many elements in serum could account for these results, we focused our validation analysis on potential upstream miRNA candidates. *In silico* analysis revealed candidate miRNAs which could be responsible for driving the DE gene profiling results. Four of the top miRNA candidates were further probed for validation via qPCR on serum samples from a larger cohort of patients. We found that 2 out of the 4 were differentially expressed in serum from patients with metastatic versus local disease.

Metastasis is a culmination of factors from both the tumor and microenvironment. Factors provided by the primary tumor can trigger inflammation, activate the endothelium, and induce bone marrow-derived cells to enter the bloodstream and mobilize to premetastatic sites. Inflammatory cells also have an inherent plasticity that can be directed by local tissue factors in the microenvironment [2].

The immune system’s complex role in defense against pathogens and its pro- and anti-tumorigenic functions make it a network of opposing signals. Inflammation can both prevent the spread of infection as well as promote tumor growth and metastasis. Inflammation is involved in all stages of cervical carcinogenesis, from Human Papillomavirus (HPV) infection to tumor initiation to conversion to metastatic disease. HPV escape from immune surveillance and establishment of long-term viral persistence is essential for the development of cervical cancer. Thus, this immune response to infection could promote tumorigenesis and possible metastasis, as suggested by our transcriptional signatures. Cervical tumors are exposed to various local inflammatory insults from bacteria in the vagina in addition to the genetic alterations caused by viral oncogenes.

This study identified differentially enriched pathways involved in cellular responses to interferon-alpha, LTA, and *S*. *aureus*. Infection with *S*. *aureus* can trigger skin inflammation and modulate immune responses. LTA, a major constituent of the cell wall of gram-positive bacteria such as *S*. *aureus*, allows the pathogen to be immunologically recognized by binding to Toll-like and platelet-activating factor receptors [16]. In a transgenic model of skin cancer induced by HPV 16 oncogenes, infiltrating CD4^+^ T cells detected in malignant lesions were predominantly reactive toward *S*. *aureus* antigens and not HPV oncogenes. This suggests that *S*. *aureus* infection in skin lesions may evoke an inflammatory response that enhances malignant progression [17]. In a genome-wide association study, Chen *et al*. identified germline variants in women with cervical cancer that were present in genes associated with the *S*. *aureus* pathway [18]. Like skin cancers, cervical cancers are colonized by bacteria found within the vagina, and further examination of the effects of *S*. *aureus* in cervical cancer metastasis warrants further investigation.

IL-10 was the most significant upstream feature in our DE analysis, with a z-score of −2.094. IL-10 is produced by T and B lymphocytes, myeloid antigen presenting cells, and epithelial cells, and it can stimulate or inhibit the immune system depending on disease state and immune response phase. However, little is known about the role of IL-10 in metastatic cervical cancer. Studies have found a correlation between HPV infection and serum IL-10 levels in local tissue [19]. In fact, two studies found an increase in serum IL-10 associated with various stages of cervical cancer [20,21]. Moreover, immature stromal dendritic cells expressing IL-10 are more numerous in cervical cancer than in normal cervix and cervical intraepithelial neoplasia [19]. IL-10 has been thought to be predominately immunosuppressive and thus tumor-promoting, but recent work has provided evidence for IL-10 mediated reduction in tumor growth and metastasis by preventing inflammatory cytokine production in mice transplanted with colon cancer cells [22]. IL-10 can also expand CD8+ tumor- infiltrating lymphocytes, promoting their killing activity. Interestingly, this effect is inhibited by complement signaling, which is activated in plasma of cancer patients [23].

Immunoglobulins are antibodies that are primarily produced by differentiated B lymphocytes, but over the last decade cytoplasmic and secreted forms of immunoglobulin production have been found in human epithelial cancers [24]. Liao *et al*. showed that immunoglobulin G (IgG) is expressed in cancer cells of epithelial lineage and that cells with higher IgG are more invasive and metastatic [24]. Wang *et al*. provided evidence that IgG is a positive regulator of lipopolysaccharide-induced pro-inflammatory cytokine production in cervical cancer cells because it binds to the Toll-like receptor 4 (TLR4), enhancing its expression. Thus, IgG might promote cervical cancer proliferation by enhancing TLR4 signaling [25].

Many studies have measured miRNAs levels in serum because miRNAs are durable biomarkers [26, 27]. Our approach was different in that we identified candidates by their ability to induce DE genes found in our reporter PBMC. We then selected specific candidate miRNAs for validation in a larger sample of sera from women with local and metastatic cervical cancer. miRNAs can deliver content-dependent pro- and anti-tumorigenic activity and show variation between intracellular and extracellular departments [27]. In fact, a recent paper showed that the abundance of miRNAs is greatly influenced by E6 and E7 oncogene expression in cervical cancer cell lines [28]. Here, we measured serum levels of the 4 candidate miRNAs (miR-23a-3p, miR-23b-3p, miR-193b-5p, and miR-944) and found that the expression of miR-23b was significantly reduced and the expression of miR-944 was significantly increased in women with metastatic versus local cervical cancer. miR-23b-3p exhibits anti-tumor activity by regulating multiple signaling pathways involved in cell differentiation and cellular immune responses [29]. miRNA-23b has been shown to be downregulated in cervical cancer tissue and upregulated when E6 and E7 oncogenes are silenced in cell line studies [28]. Furthermore, miRNA-944 expression has been shown to promote cell proliferation, migration, and invasion in human cervical cancer cells, supporting our finding that its expression is enhanced in serum from patients with metastatic disease [30].

The major limitation of our study is its small sample size, as it examined 10 patients in the discovery phase. Also, relying on cryopreserved PBMCs from a single donor provides a uniform assay platform, but it is unlikely to capture all possible transcriptional signals given the genetic variability among individuals. Moreover, use of an indirect cell-based assay prevented us from identifying and measuring all upstream immune factors. The serum samples were also stored at -80° Celsius for varying lengths of time because they were collected at diagnosis. However, miRNAs are very stable biomarkers and have been shown to vary little after long-term storage or with mode of storage, which is why we explored these serum factors further in this study [31]. Moreover, while we cannot easily test the effects of storage time on a single sample, we do know from prior studies done at our laboratory that samples collected a decade apart in patients with type 1 diabetes and stored at -80° Celsius produce highly correlative transcriptional signatures [4,5].

In summary, we provide evidence that a PMBC reporter assay can distinguish local from metastatic disease by measuring the induction of DE genes triggered by sera from women with cervical cancer. Expanding this study to a larger patient population would offer additional insights into the immunosuppressive and proliferative pathways that may contribute to cervical cancer progression. As serum contains many types of circulating factors, future work could examine other upstream factors in addition to miRNAs. Also, because current treatment for metastatic cervical cancer is not curative, identification of additional inflammatory pathways that contribute to metastasis is critical for more efficient cancer diagnosis and treatment. Finally, future prospective studies are required to further validate the contribution of miRNAs found in this study to cervical cancer metastasis.

## Supporting information

S1 Table GO and KEGG enrichment results. (CSV)

S1 Fig Graphical abstract of the experiment. (TIF)
