# Correction: Identification of a serum-induced transcriptional signature associated with metastatic cervical cancer

**DOI:** 10.1371/journal.pone.0193687

**Published:** 2018-02-23

**Authors:** Anna Palatnik, Shuyun Ye, Christina Kendziorski, Marissa Iden, Jessica S. Zigman, Martin J. Hessner, Janet S. Rader

S1 Table is incomplete. Please view the complete S1 Table below.

## Supporting information

S1 Table GO and KEGG enrichment results. (XLSX)
