# Supplementary figures and images for: Identification of a serum-induced transcriptional signature associated with metastatic cervical cancer
Source: PLoS One. 2017 Aug 30;12(8):e0181242. doi: 10.1371/journal.pone.0181242 (PMC5576712; doi:10.1371/journal.pone.0181242)

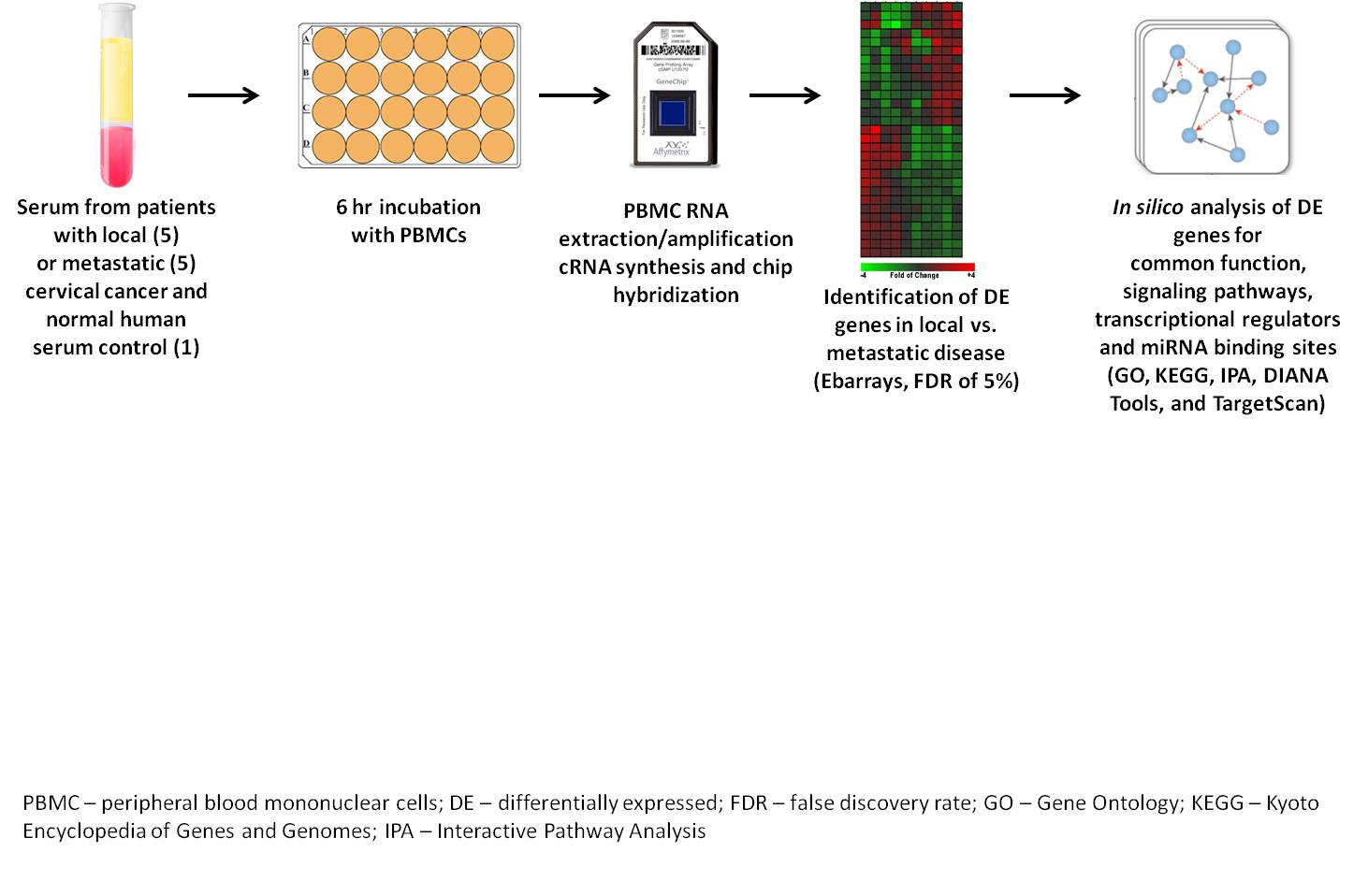

Supplement: S1 Fig — (TIF) [file pone.0181242.s002.tif]
